# Presence of Polyketide Synthase (PKS) Gene and Counterpart Virulence Determinants in *Klebsiella pneumoniae* Strains Enhances Colorectal Cancer Progression In-Vitro

**DOI:** 10.3390/microorganisms11020443

**Published:** 2023-02-09

**Authors:** Christina Parvinder Kaur, Thevambiga Iyadorai, Cynthia Sears, April Camilla Roslani, Jamuna Vadivelu, Chandramathi Samudi

**Affiliations:** 1 Department of Medical Microbiology, Faculty of Medicine, University of Malaya, Kuala Lumpur 50603, Malaysia; 2 Department of Medicine, Johns Hopkins University School of Medicine, Baltimore, MD 21205, USA; 3 Department of Oncology, Sidney Kimmel Comprehensive Cancer Center, Johns Hopkins University School of Medicine, Baltimore, MD 21278, USA; 4 Department of Surgery, University of Malaya, Kuala Lumpur 59100, Malaysia; 5 University of Malaya Cancer Research Institute, Kuala Lumpur 50603, Malaysia

**Keywords:** *K. pneumoniae*, colorectal cancer, polyketide synthase (PKS), colibactin, hypervirulent *K. pneumoniae* (hvKp), pyogenic liver abscess (PLA), siderophores, biofilm, string test, antibiotic resistance, electrical cell-substrate impedance sensing (ECIS)

## Abstract

*Klebsiella pneumoniae* (*K. pneumoniae*) colonizes the human gut and is a causative factor of pyogenic liver abscess (PLA). Retrospective studies conducted on *K. pneumoniae* PLA patients revealed subsequent CRC development in later years of their life with increasing prevalence of these strains harbouring polyketide synthase (PKS) genes. To our knowledge there are no known studies directly implicating *K. pneumoniae* with CRC to date. Our aims are to characterize *K. pneumoniae* isolates from CRC patients and investigate its effects on cell proliferation in vitro. *K. pneumoniae* isolates were characterized by screening virulence genes including polyketide synthase (PKS), biofilm assay, antibiotic susceptibility, and string test to determine hypervirulent (hvKp) strains. Solubilised antigens of selected *K. pneumoniae* isolates were co-cultured with primary colon cell lines and CRC cell lines (Stage I-IV) for 48 h. The enhancement of proliferation was measured through MTT and ECIS assay. Twenty-five percent of *K. pneumoniae* isolates were PKS-positive out of which 50% were hvKp strains. The majority of the isolates were from the more virulent serotype of K1 (30%) and K2 (50%). PKS-positive *K. pneumoniae* isolates did not possess genes to confer carbapenem resistance but instead were more highly associated with siderophore genes (aerobactin, enterobactin, and yersiniabactin) and allantoin metabolism genes (*allS, allS2*). Cell proliferation in primary colon, SW1116 (Stage I), and SW480 (Stage II) CRC cell lines were enhanced when co-cultured with PKS-positive *K. pneumoniae* antigens. ECIS revealed enhanced cell proliferation upon recurrent antigen exposure. This demonstrates the possible role that PKS-positive *K. pneumoniae* has in exacerbating CRC progression.

## 1. Introduction

*Klebsiella pneumoniae* (*K. pneumoniae*) is a Gram-negative bacterium of the Enterobacteriaceae family that colonizes human mucosal surfaces such as the nasopharynx and the gastrointestinal tract (GIT) [1,2]. *K. pneumoniae* has been an aetiological agent in a number of diseases such as hospital acquired infections, bacteraemia, endocarditis, pneumonia, as well as pyogenic liver abscess (PLA) [3,4]. In recent years, the incidence rate of *K. pneumoniae* infections worldwide has risen dramatically due to the emergence of multi-drug resistant (MDR) as well as extremely drug resistant (XDR) strains [5]. The lack of new antibiotics in the pipeline has also dampened efforts to curb and contain the infection. In addition to that, the development and dissemination of hypervirulent *K. pneumoniae* strains (hvKp) among Asian countries have also caused a peak in PLA cases being reported, especially in Taiwan and China, where *K. pneumoniae* has exceeded *Escherichia coli* (*E. coli*) as the most common pathogen isolated from PLA patients [6]. Increasing reports indicate the rise of hvKp strains being isolated from PLA patients with the majority of these strains belonging to the K1 and K2 serotype that are known to confer greater virulence. The hvKp differs from the classical *K. pneumoniae* (cKp) due to its increased virulence and ability to cause more severe infections compared to the latter. The hvKp strains are notorious for their biofilm forming properties as well as efficient iron uptake systems and siderophore production. These are some of the virulence factors that enhance the survival of *K. pneumoniae* within the host through evasion of host immune defences and its ability to sequester iron, allowing it to persist within the host. Furthermore, *K. pneumoniae* is known to harbour the polyketide synthase (PKS) island within its genome that produces colibactin genotoxin. This phenomenon has also been observed in *E. coli*, where reports demonstrated colibactin genotoxin’s ability to cleave host DNA double strands bringing about cell cycle arrest as well as DNA damage and mutations which initiates tumorigenesis [7]. This is pertinent, as an 11-year retrospective study conducted on PLA patients revealed that *K. pneumoniae* PLA patients had approximately three times higher risk of developing colorectal cancer (CRC) years after a PLA infection compared to non-*K. pneumoniae* PLA patients [8,9,10,11,12]. The common notion indicates PLA as a precursor disease and a risk factor of CRC development which is the second most common cancer diagnosed worldwide irrespective of gender. However, a case study by Li et al., of a 65-year-old man with no known chronic illnesses and surgical history, was suffering from hepatic portal venous gas (HPVG) demonstrated radiologically as pockets of gas that are formed as a by-product of *K. pneumoniae* metabolism that enter the portal venous system. [13]. The blood culture test conducted further validated the presence of *K. pneumoniae* and antibiotic treatment was implemented. Furthermore, upon treating the patient with empiric antibiotic therapy (sulbenicillin 8 g/day and metronidazole 2 g/day), there was a reduction in gas accumulation due to the inhibited reproduction of *K. pneumoniae*. The same patient was then diagnosed with colon cancer (TNM staging: T4bN2bM0) after a few days.

*K. pneumoniae* is a common colonizer of the GIT, especially the colon, and possibly enters the hepatic portal veins which receive blood from the GIT through the network of mesenteric veins. The inferior mesenteric vein transports blood from the colon and rectum to the hepatic portal vein which sends it to the liver before being transported back to the heart. It could also be postulated that *K. pneumoniae* enters the liver through this similar pathway to cause PLA.

Although numerous studies have performed characterization of *K. pneumoniae* isolated from PLA patients, there are still gaps and chinks in the armour that directly link *K. pneumoniae* with CRC without PLA being a precursor condition. Therefore, this study aims to characterize the virulence genes as well as the prevalence of PKS-positive *K. pneumoniae* isolates obtained from CRC patients attending University Malaya Medical Centre (UMMC) and to determine their role in CRC progression in vitro.

## 2. Materials and Methods

### 2.1. Isolation of K. pneumoniae from Clinical Specimens

*K. pneumoniae* were isolated from faecal, tumour, and tissue biopsy specimens of CRC patients and healthy individuals attending UMMC upon obtaining ethical approval from UMMC Medical Ethics Committee based on the Declaration of Helsinki 1975 (Ethics Reference Number: 943.18). In this study, 43 CRC patients and 72 healthy individuals attending UMMC for routine colonoscopy screening and who had colon or rectal resection between March 2014 to August 2015 consented to participate. All clinical specimens were processed, isolated, and cultured in the Department of Medical Microbiology, Faculty of Medicine, University of Malaya. In order to isolate pure colonies of *K. pneumoniae*, the specimens were homogenized and cultured in Luria Bertani broth overnight and streaked onto MacConkey Agar. All mucoidal colonies were subjected for identification of *K. pneumoniae* isolates, performed using Bruker Biotyper MALDI-TOF MS system according to manufacturer’s instructions. Additionally, species identification was further confirmed through 16s rRNA gene sequencing. Pure colony *K. pneumoniae* cultures were preserved in 30% glycerol stocks and stored in −80 °C until further use.

### 2.2. Maintenance of CRC Cell Lines

A total of 4 human CRC cell lines derived from various CRC stages (Stage I to IV) as well as 1 primary colon cell line were used in this study. Human primary colon cell (ABM, Cat. No: T4056) was used as a non-CRC in vitro model. SW 1116 (ATCC ^®^ CCL-233), SW 480 (ATCC ^®^ CCL-228), HT 29 (ATCC ^®^ HTB-38), HCT 116 (ATCC ^®^ CCL-247) were used as stage I-IV derived cell lines respectively. All cell lines were grown in RPMI 1640 and supplemented with 10% Fetal Bovine Serum (FBS) and 1% each of Penicillin-Streptomycin, L-Glutamine, and Amphotericin B at 37 °C with 5% CO_2_.

### 2.3. Genomic DNA Extraction

*K. pneumoniae* DNA were extracted according to manufacturer’s protocol using the Qiagen DNeasy Blood & Tissue Kit. The purity and concentration of extracted DNA were measured and quantified using the IMPLEN NanoPhotometer. The DNA purity of 1.8–2.0 (A_260_/A_280_ ratio) was used for subsequent experiments. Extracted DNA that was not utilized immediately were stored in aliquots at −20 °C. The positive and negative controls used were *K. pneumoniae* ATCC 13883 extracted DNA and nuclease free water respectively.

### 2.4. PCR Detection of Virulence Genes including PKS Colibactin Genotoxin

A total of 41 primer sets were used to screen and detect *K. pneumoniae* virulence genes which included PKS colibactin gene, *K. pneumoniae* serotypes (K1(*K1-magA*), K2 (*K2wzy*), K5 (*K5wzx*), K20 (*wzyK20*), K54 (*wzxK54*), K57 (*wzyK57*)) as well as Carbapenem genes (OXA-48 (*blaOXA-48*), NDM-1 (*blaNDM-1*), KPC (*blaKPC*), IMP (*blaIMP*), and VIM (*blaVIM*)). Previously published primers that were used in this study along with the length of the expected PCR product are listed in Supplementary S1. Briefly, the counterpart virulence genes that were screened are Adhesins (*mrkD1, mrkD2, fimH, kpn*), Enterobactin siderophores (*entB, entB1*), Ferric aerobactin receptor (*iutA, iutA1,iucB*), Aerobactin siderophore, Yersiniabactin siderophore (*ybtA, ybtS*), Salmochelin catecholate receptor (*iroN, iroNB*), *Klebsiella* iron uptake systems (*kfu, kfuBC*), Genotoxins(Colibactin (*clbB*), Haemolysin (*hlyA*), Cytotoxic necrotizing factor (*cnf*)), Capsule synthesis (K2 serotype capsule synthesis (*k2A*), Fucose capsule synthesis (*wcaG*), Lipid polysaccharide (LPS) synthesis (*wabG*), outer membrane lipoprotein (*ycfM*), Uridine diphosphategalacturonate 4-epimerase (*uge*), Regulator of mucoid phenotype A (*rmpA1, rmpA2*), urease synthesis (*ureA*), and Allantoin metabolism (*allS, allS2*). The cycling conditions for the detection of PKS colibactin gene were initial denaturation at 95 °C for 10 min, denaturation at 94 °C for 45 s, annealing at 54 °C for 45 s, and extension at 72 °C for 1 min. This was repeated for 30 cycles. The amplified PCR products were separated and visualized through gel electrophoresis at 120 V for 2 h in 1.8% (wt/vol) agarose gel containing SYBR Safe DNA Gel Stain (Invitrogen). Cycling conditions used for the multiplex PCR along with the primer grouping will be made available upon request.

### 2.5. Antibiotic Susceptibility Testing (AST)

A total of 40 clinical *K. pneumoniae* isolates and 1 *K. pneumoniae* ATCC 13883 strain were screened against a panel of 29 antibiotics from 16 antimicrobial categories using Kirby-Bauer disc diffusion method and broth microdilution methods were based on previously established protocol by Wiegand et al. and Hudzicki et al. [14,15] according to Clinical and Laboratory Standards Institute (CLSI) [16] and European Committee on Antimicrobial Susceptibility Testing (EUCAST) [17] antibiotic breakpoints guidelines, respectively. The antibiotics used are listed in Table 1.

### 2.6. Biofilm Assay

This assay was performed according to previously established protocols by Singh et al. and Emery et al. [18,19] with slight modifications. Single colony *K. pneumoniae* isolates were inoculated in 5 mL of trypticase soy broth for 24 h at 37 °C. The inoculum was adjusted to 0.5 MacFarland standard (equivalent to 1.5 × 10^8^ CFU/mL). 200 µL of the adjusted inoculum were seeded into wells of the 96-well flat bottom plates and incubated at 37 °C for 24 h. Inoculum were carefully removed, and the plates were washed three times in sterile phosphate buffered saline (PBS). The adherent layer of bacteria was heat-fixed for 20 min at 60 °C. An amount of 125 µL of 0.1% crystal violet was added to the wells and incubated at room temperature for 15 min. The plates were rinsed with sterile distilled water 3 times and excess water and dye were blotted using paper towels. An amount of 200 µL of 33% glacial acetic acid was added to the wells and incubated at room temperature for 1 h. The absorbance was measured at 570 nm and the biofilm-producing classification was determined based on the values below:

Negative control = DO_c_

Absorbance value of each strain = DO_a_

Non-adherent = DO_a_ ≤ DO_c_

Weakly adherent = DO_c_ < DO_a_ ≤ 2xDO_c_

Moderately adherent = 2xDO_c_ < DO_a_ ≤ 4xDO_c_

Strongly adherent = 4xDO_c_ < DO_a_

### 2.7. String Test

*K. pneumoniae* isolates were grown on nutrient agar plates overnight at 37 °C. A sterile inoculating loop was used to touch the surface of a single *K. pneumoniae* colony and to stretch the colonies by raising the inoculating loop to produce a mucoviscous string. The test was considered positive if the isolates were able to produce a mucoviscous string of > 5 mm in length.

### 2.8. Solubilised Antigen Extraction (Crude Whole Cell Extract)

Single colonies of *K. pneumoniae* isolates were grown separately in 200 mL of Luria Bertani (LB) broth in a conical flask at 37 °C for 18 h with shaking at 180 rpm. After incubation, the grown inoculum was transferred to 50 mL conical tubes and centrifuged at 18,000× *g* for 30 min at 4 °C. The supernatant was discarded, and the bacterial cell pellet was washed twice in cold sterile PBS and resuspended in sterile ice-cold PBS at a concentration of 1 g wet weight per ml. The suspension was sonicated to rupture the bacterial cell wall for 15 min at 4 °C with 40 s burst at 50% power setting. The sonicated suspension was then briefly vortexed at top speed and allowed to stand overnight at 4 °C to allow complete solubilisation of the *K. pneumoniae* bacterial antigens. The suspension was sequentially filtered through 0.45 µm and 0.20 µm syringe filters. An amount of 10 µL of the filtered solubilised antigen was spread onto nutrient agar and incubated at 37 °C overnight to determine no viable bacterial cells were present. The whole bacterial antigens extracted are protein in nature and will be used as such. Further purification and determination of the antigen properties were not performed in this study. From here on, the *K. pneumoniae* solubilized antigens will be referred to as bacterial antigens. The protein concentration was estimated using the Bradford’s method and the extracts were stored in aliquots at −20 °C until further use.

### 2.9. Enhancement of Cell Proliferation (In-Vitro)

Human primary and CRC cell lines were seeded into wells of 96-well flat bottom plates at a density of 500 cells/100 µL per well (5.0 × 10^3^/mL). The cells were incubated at 37 °C with 5% CO_2_ overnight to allow the cells to adhere. MTT assay was performed to determine the appropriate concentration of bacterial antigen to be used (data not shown). After incubation, 10 µL of prepared bacterial antigen was added to the well at a concentration of 500 µg/mL (~50 µg/mL final concentration within well). The optimal concentration of bacterial antigen to be used was determined through MTT assay. 10 µL of EGF at a final well concentration of 20 ng/mL and 10 µL of sterile PBS were used as positive and negative controls respectively. All conditions were in triplicates. The *K. pnuemoniae* bacterial antigens used were obtained from 4 main isolate groups, namely PKS-positive of CRC, PKS-positive of healthy, PKS-negative of CRC, and PKS-negative of healthy origin. The cells were then incubated for 48 h at 37 °C with 5% CO_2_. To measure the enhancement of cell proliferation, 10 µL of 5 mg/mL (final concentration) MTT dye were added to all wells and incubated for 4 h at 37 °C with 5% CO_2_. After incubation, the supernatant was carefully aspirated so as to not disturb the cell monolayer and 100 µL of DMSO were added to the wells and kept on a rocking platform for 10 min to solubilise the MTT dye. The absorbance was read on a microtitre plate reader at 570 nm. The enhancement of cell proliferation was read after normalizing the values obtained from the negative control wells.

### 2.10. Electrical Cell-Substrate Impedance Sensing (ECIS)

The ECIS array measures the increase in electrical impedance that directly corresponds to the increase in cell proliferation. The ECIS array contains gold electrodes at the bottom of the wells that are able to detect the growth of cells. As the cells proliferate and cover the electrode, the impedance increases until confluency is achieved. Briefly, CRC cells were seeded at a density of 50,000 cells per well in 400 µL of medium in 8 well plates with 10 gold electrodes (8W10E PET, Ibidi, Gräfelfing, Germany). The impedance was measured using the ECIS model Zθ (Z Theta) (Applied Biophysics, US, Troy, MI, USA). The cells were incubated for a period of 6 days at 37 °C with 5% CO_2_ with medium renewal at day 3 as well as antigen introduction at days 1 and 3.

### 2.11. Statistical Analysis

The IBM Statistical Package for Social Science (SPSS) software version (26.0) was used for statistical analysis. A Pearson chi-square was used to find the correlation between the relationship of PKS-positive and hypervirulent isolates against the screened virulence genes. A *p* value less than 0.05 was considered to be statistically significant.

## 3. Results

### 3.1. PKS-Positive K. pneumoniae Isolates Harboured More Virulence Genes Compared to PKS-Negative Isolates

#### 3.1.1. Presence of Colibactin (PKS) and Capsular Serotype

After screening 43 CRC patients and 72 healthy individuals, only 40 individuals (15 CRC and 25 healthy individuals) harboured *K. pneumoniae*. Out of the 40 clinical isolates, 25% (10/40) of the isolates harboured the PKS colibactin genotoxin gene and a K-antigen capsular serotyping screening revealed that 14/40 of the isolates could be assigned to the six most prevalent serotypes (K1, K2, K5, K20, K54, K57) of *K. pneumoniae*. The most virulent serotypes that have been associated with hypervirulence are the K1 and K2 serotype. Screening of the ten PKS-positive isolates showed that three (30%) of these isolates belonged to the K1 serotype, five (50%) were K2, one (10%) isolate was K5 and the remaining one isolate did not belong to the any of the serotypes screened. On the other hand, all three of the PKS-positive K1 serotype isolates were obtained from clinical specimens of CRC patients and the remaining seven PKS-positive isolates were obtained from healthy individuals attending UMMC for routine medical examinations. There was no statistical difference observed among the PKS-positive *K. pneumoniae* strains isolated from CRC and healthy individuals. Further assessment of other virulence genes is vital to understand if other virulence factors play a role in increasing the pathogenicity of PKS-positive *K. pneumoniae*. The presence of virulence genes was determined solely based on a PCR and sequencing of biosynthetic gene clusters will be required to determine the functionality of the virulence genes. Table 2 shows the summary of the serotypes and colibactin gene detected from *K. pneumoniae* isolates.

#### 3.1.2. Siderophores and Iron Uptake Systems

**Figure 1:**
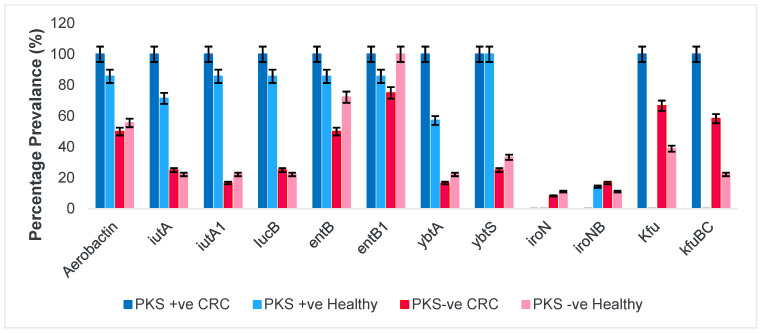
Presence of siderophores and iron uptake systems among *K. pneumoniae* isolates. Aerobactin siderophore, ferric aerobactin receptor (*iutA, iutA1,iucB*), enterobactin siderophores (*entB, entB1*), Yersiniabactin siderophore (*ybtA, ybtS*), salmochelin catecholate receptor (*iroN, iroNB*) and *Klebsiella* iron uptake systems (*kfu, kfuBC*).

Iron is an important element that is required for bacterial survival and pathogenesis. Although, iron is abundantly found within the host, most of the iron are bound to host proteins such as haemoglobin and transferrin. To overcome this, *K. pneumoniae* sequesters iron from host bound proteins through the secretion of siderophore molecules which are high-affinity iron-chelating agents. The four groups of siderophores are aerobactin, enterobactin, salmochelin, and yersiniabactin, which all play different roles in establishing an infection within the host. In this study, PKS-positive isolates harboured genes responsible for the production of aerobactin, enterobactin, and yersiniabactin siderophores. A key notable difference observed was the lack of the salmochelin (*iroN, iroNB*) siderophore gene among PKS-positive isolates from CRC patients and healthy individuals as well as lack of iron uptake systems (Kfu, KfuBC) among PKS-positive isolates from healthy individuals. In addition to that, PKS-negative isolates predominantly possessed genes conferring production of enterobactin siderophore. Similarly, the salmochelin siderophore was also found in approximately 17% of PKS-negative isolates from CRC patients and 11% of PKS-negative isolates from healthy individuals. Figure 1 shows the distribution and prevalence of siderophores as well as the iron uptake systems of the isolates.

#### 3.1.3. Regulator of Mucoid Phenotype and Accessory Genes

**Figure 2:**
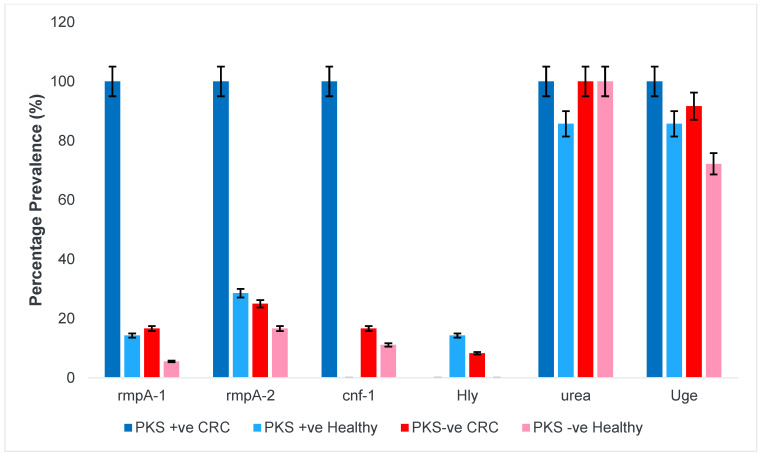
Prevalence of regulator of mucoid phenotype and accessory genes among *K. pneumoniae* isolates. Regulator of mucoid phenotype A (*rmpA1, rmpA2*), cytotoxic necrotizing factor (*cnf*)), haemolysin (*hlyA*), urease synthesis (*urea*) and uridine diphosphategalacturonate 4-epimerase (*uge*) gene.

The regulator mucoid phenotype gene (rmpA) is responsible for enhancing extracellular capsule synthesis resulting in increased mucoviscosity of the isolates. The presence of the rmpA gene is also one of the virulence determinants for *K. pneumoniae* hypervirulent strains. All the PKS-positive isolates from CRC patients had the rmpA gene. Additionally, the cytotoxic necrotizing factor-1 (cnf-1) works in concert with the colibactin gene to modulate the cell cycle that contribute to tumorigenesis and hyperproliferation. This gene was also found in all PKS-positive isolates of CRC origin. However, the cnf-1 gene was not present in any of the PKS-positive strains isolated from healthy individuals and therefore it would be interesting to observe how it affects the enhancement of cell proliferation. Interestingly, the haemolysin gene (hly) was found in a low abundance of 14% and 8% among PKS-positive from healthy individuals and PKS-negative isolates from CRC patients, respectively. The two fundamental genes that are involved in colonization and infection for *K. pneumoniae* are the uge and urea gene. The uge gene plays a role in the initial colonization and infection of the gastrointestinal mucosa whereas the urea gene aids in driving the persistence of infection by overcoming the gastrointestinal stress through the utilisation of nitrogen for protein synthesis. Figure 2 shows the prevalence of genes responsible for mucoid variants, genotoxins and capsule synthesis. 

#### 3.1.4. Fimbriae and Adhesins

**Figure 3:**
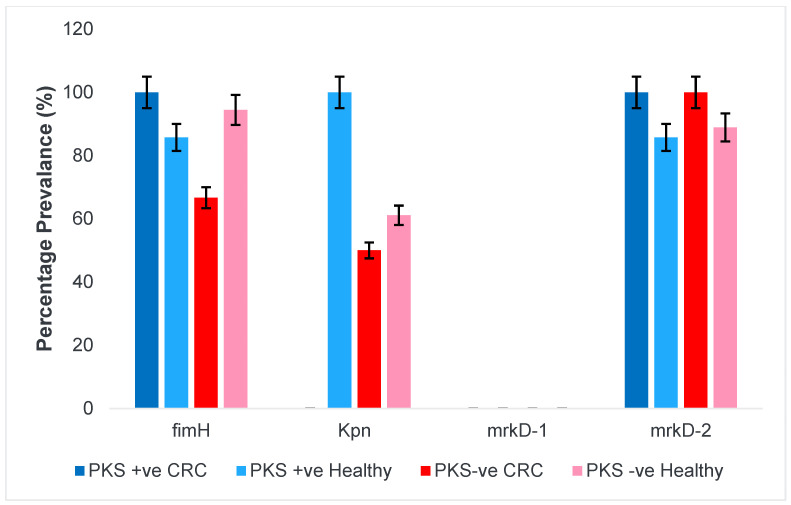
Prevalence of fimbriae and adhesins among *K. pneumoniae* isolates. Adhesins (*mrkD1, mrkD2, fimH, kpn*).

Fimbriae and adhesins are vital for the attachment and colonization on the colonic mucosa. The kpn gene which is responsible for type 1 fimbriae-like adhesin is lacking among PKS-positive isolates from CRC patients. However, it is found in 100%, 50%, and 61% of PKS-positive (healthy), PKS-negative (CRC), and PKS-negative (healthy) isolates respectively. None of the isolates possessed the mrkD-1 gene which is a type 3 fimbriae but was compensated by the presence of other adhesins and fimbriae. Figure 3 shows the prevalence of genes responsible for presence of fimbriae and adhesins.

#### 3.1.5. Allantoin Metabolism and Lipopolysaccharide

**Figure 4:**
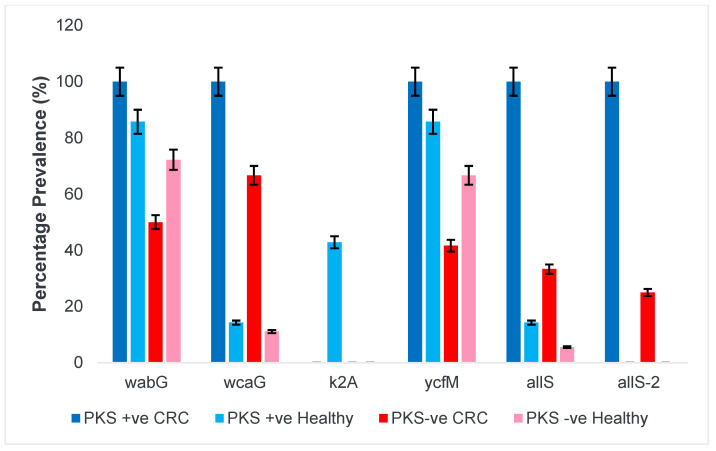
Prevalence of allantoin metabolism and lipopolysaccharide among *K. pneumoniae* isolates. Lipid polysaccharide (LPS) synthesis (*wabG*), fucose capsule synthesis (*wcaG*), capsule synthesis (K2 serotype capsule synthesis (*k2A*), outer membrane lipoprotein (*ycfM*), and allantoin metabolism (*allS, allS2*).

Allantoin metabolism is vital for the utilisation of allantoin as a main source of nitrogen and carbon. It is also an important hallmark feature to determine the severity of infection. *Escherichia coli* (*E. coli*) can only use allantoin as a source of nitrogen. However, *K. pneumoniae* has the ability to metabolize allantoin and utilize it as a source of carbon and nitrogen. This allows it to persist within the host for a prolonged time and survive under stressful conditions. Figure 4 shows the prevalence of genes associated with allantoin metabolism and lipopolysaccharide synthesis.

A correlation analysis was performed to determine the relationship of PKS-positive and hypervirulent isolates against the screened virulence genes (Table 3). The analysis revealed there were strong positive correlations (r = 0.7–1.0) between *wabG, ycfM, entB, entB1, fimH, iutA, iutA1, iucB, rmpA1, rmpA2, kfuBC, kfu, allS*, and *allS2* (having a correlation significance at 0.01 level). We also observed that the presence of the pks gene is correlated with the hypervirulent *K. pneumoniae* strains as previously described. Additionally, these Pks-positive hypervirulent *K. pneumoniae* isolates possess a high correlation between the *ycfM, ybtS, ybtA*, (yersiniabactin) *iutA, iutA1, aerobactin, iucB*, (aerobactin) *rmpA1, rmpA2* (regulator of mucoid), and *k2A* (K2 capsule) genes.

### 3.2. Carbapenem Resistance Genes and Its Correlation with Antibiotic Susceptibility Testing (AST)

Antibiotic resistance is a worldwide threat, particularly in clinical settings where growing cases of MDR, XDR, and Pandrug resistance (PDR) infections are being reported. With the rise of hvKp strains being reported in Asian countries it is important to contain the spread of these isolates and prevent the emergence of MDR-hvKp strains prevailing. An isolate is considered to be MDR when it is non-susceptible to ≥1 agent in ≥3 antimicrobial categories. An XDR isolate is defined as non-susceptible to ≥1 agent in all but ≤2 categories and a PDR isolate is non-susceptible to all antimicrobial agents [20].

After screening fourty clinical *K. pneumoniae* isolates and one *K. pneumoniae* ATCC 13883 strain, seven clinical isolates were identified as MDR strains, all of which were PKS-negative. PKS-positive *K. pneumoniae* isolates were only resistant to the penicillin group of antibiotics (ampicillin (6/10) and amoxycillin (10/10)). On the other hand, PKS-negative *K. pneumoniae* conferred antibiotic resistance to amoxycillin (30/30), ampicillin (27/30), chloramphenicol (6/30), tetracycline (4/30), trimethoprim-sulfamethoxazole (4/30), doxycycline (3/30), levofloxacin (3/30), tigecycline (2/30), amikacin (1/30), amoxicillin-clavulanic acid (1/30), and norfloxacin (1/30). AST results are tabulated in Table 4.

Additionally, none of the PKS-positive isolates carried the genes that confer resistance towards carbapenems. However, three PKS-negative isolates carried the *bla*_OXA-48_ gene. Of these strains, two out of three were isolated from healthy individuals and one strain was isolated from a CRC patient. The summary of isolates harbouring carbapenem resistance are shown in Table 5.

### 3.3. Presence of PKS Gene Improves Biofilm-Forming Capabilities of K. pneumoniae Isolates

**Figure 5:**
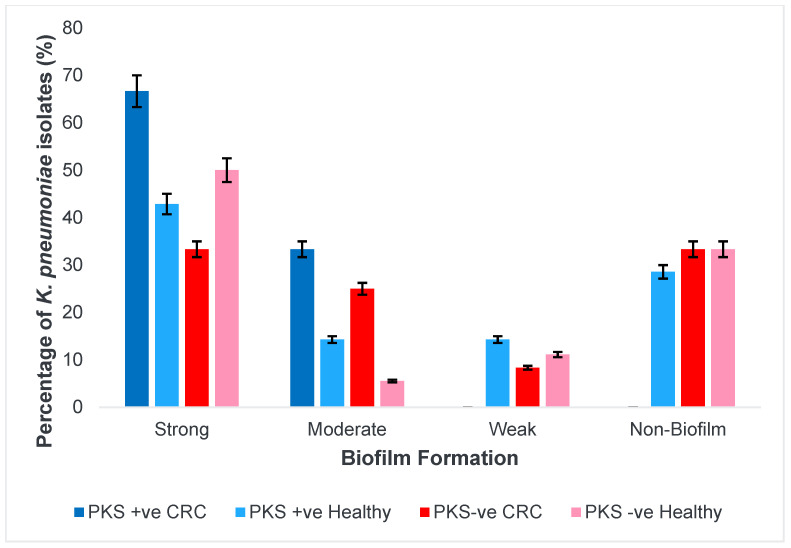
Biofilm forming capabilities of *K. pneumoniae* isolates.

**Figure 6:**
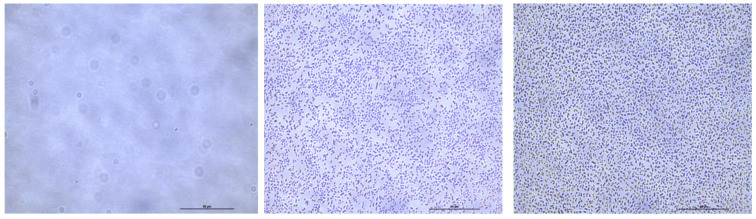
Crystal violet Staining of *K. pneumoniae* Biofilm.

*K. pneumoniae* biofilm-forming capabilities are a cause of the majority of indwelling catheter infections among patients. In this study, 18 out of the 40 isolates screened were strong biofilm producers. Among this group, five isolates were PKS-positive strains and out of which three isolates were obtained from CRC patients. There were six moderate biofilm producers with two PKS-positive isolates, one of which came from a CRC patient. Four isolates were weak biofilm producers having only one PKS-positive isolate obtained from a healthy individual. The remaining 12 out of the 40 isolates screened were non-biofilm producers out of which two PKS -positive isolates were obtained from healthy individuals. Figure 5 shows the breakdown of isolates based on their biofilm forming capabilities, PKS status, and isolate origin and the demonstration of biofilm formation was revealed through crystal violet staining in Figure 6. Overall, the PKS +ve isolates of CRC origin are strong to moderate biofilm producers. Table 6 shows the distribution of biofilm forming capabilities among *K. pneumoniae* isolates.

### 3.4. Prevalence of Hypervirulent K. pneumoniae (hvKp) Strains

**Figure 7:**
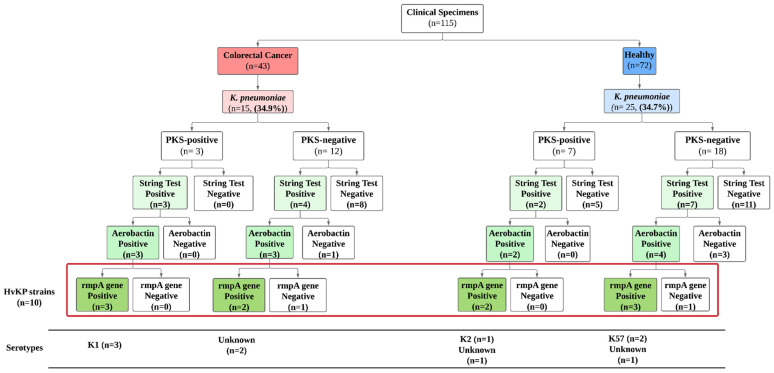
hvKp strains serotype and origin characteristics.

**Figure 8:**
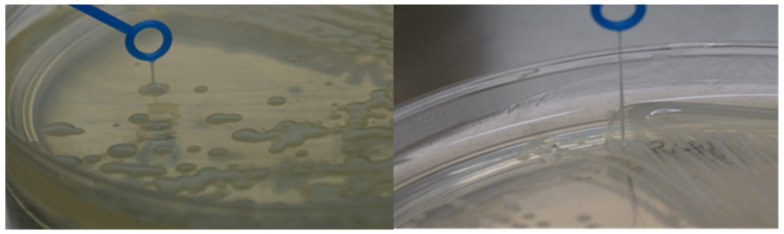
Mucoviscous string production in positive string test.

The string test alone is not a definitive test to determine the hvKp strain, and therefore it should be used in concert with the presence of the aerobactin siderophore gene as well as the rmpA gene. It is only when these three genes are present together within a single isolate of *K. pneumoniae* that the strain is considered to be a hvKp strain [21]. From the 40 *K. pneumoniae* isolates screened, 16 (40%) produced a positive string test. In these 16 isolates, 10 isolates (62.5%) were considered hvKp based on the abovementioned definition. The further breakdown of the hvKp isolates is described in Figure 7 whereas the mucoviscous string formation of the isolates is shown in Figure 8. We found the burden of *K. pneumoniae* in CRC is similar to the healthy group, with 34.8% and 34.7% prevalence, respectively (Figure 7). The prevalence of PKS-positive strains among the CRC and healthy groups was 20% (3/15) and 28% (7/25), respectively. However, alarmingly, all the three PKS-positive *K. pneumoniae* isolates from the CRC patients were hypervirulent strains belonging to the K1 serotype. Whereas only two PKS-positive *K. pneumoniae* strains were found to be hypervirulent among the healthy group, with one isolate belonging to the K2 serotype. This implicates that the bacterium may potentially enhance CRC progression. Nevertheless, the possible correlation of *K. pneumoniae* titers in CRC patients will certainly add value to its role as a driver bacterium and this needs further exploration. In the current study its role as a driver bacterium was evidenced through in-vitro cell proliferation assay and ECIS analysis

### 3.5. Colorectal Cancer Cell Line Proliferation Is Enhanced upon Exposure to K. pneumoniae Antigens

**Figure 9:**
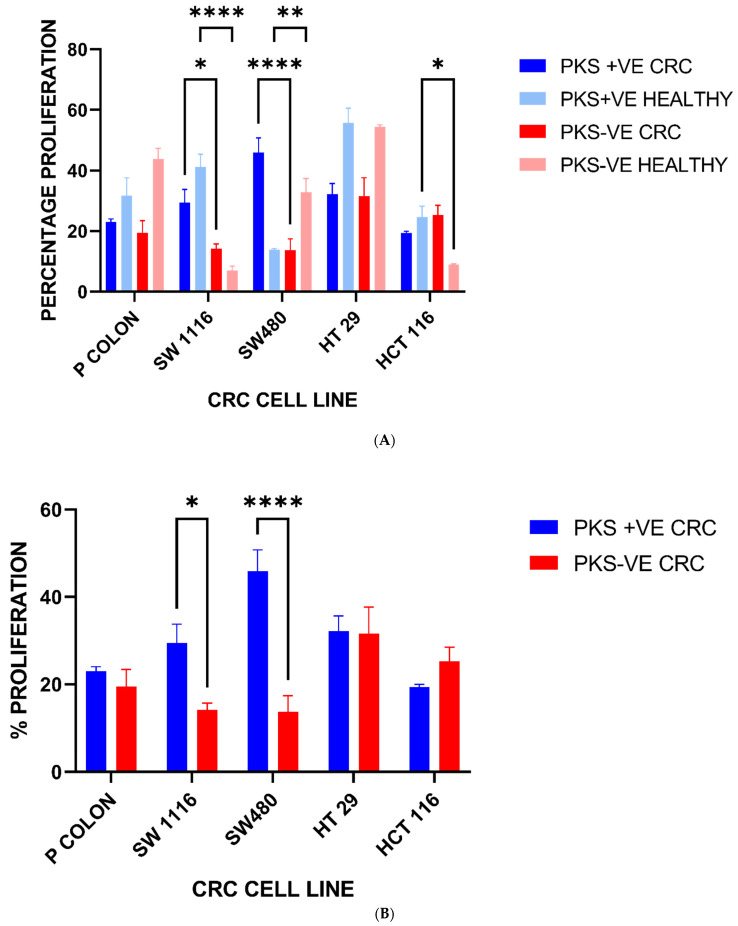
(**A**): Enhancement of cell proliferation upon exposure to *K. pneumoniae* Antigens, * = *p* <0.05, ** = *p* <0.01, **** = *p* < 0.0001. (**B**): Enhancement of cell proliferation upon exposure to *K. pneumoniae* antigens isolated from CRC patients * = *p* <0.05, **** = *p* < 0.0001.

Figure 9A shows the enhancement of the human primary and CRC cell lines upon exposure to *K. pneumoniae* antigens. The values obtained from the MTT assay were normalized against the values of the absorbance of the negative control (PBS) wells. The enhancement of cell proliferation was expressed as the percentage (%) of cell proliferation. Generally, *K. pneumoniae* antigens were able to enhance the proliferation of human primary and CRC cell lines in vitro.

However, PKS-positive isolates isolated from CRC patients had the highest enhancement of cell proliferation across all cell lines except for SW1116 (stage I), where the PKS-positive isolate from healthy patients had a slight edge. The highest enhancement of cell proliferation was observed in the SW480 (stage II) cell line. The enhancement of cell proliferation trend showed a bell-shaped curve that progressed from the human primary cell line and peaked at SW480 (stage II) after which the enhancement of cell proliferation decreased. Additionally, PKS-positive isolates showed better enhancement of cell proliferation compared to PKS-negative isolates, irrespective of isolate origin. There is some plausibility that the presence of the PKS colibactin gene along with co-virulence factors could play a role in enhancing the progression rate of CRC, especially in the normal and early stages of CRC (as observed in the primary and CRC cell line of stage I and II).

### 3.6. Persistent K. pneumoniae Infection Enhances Colorectal Cancer Progression

**Figure 10:**
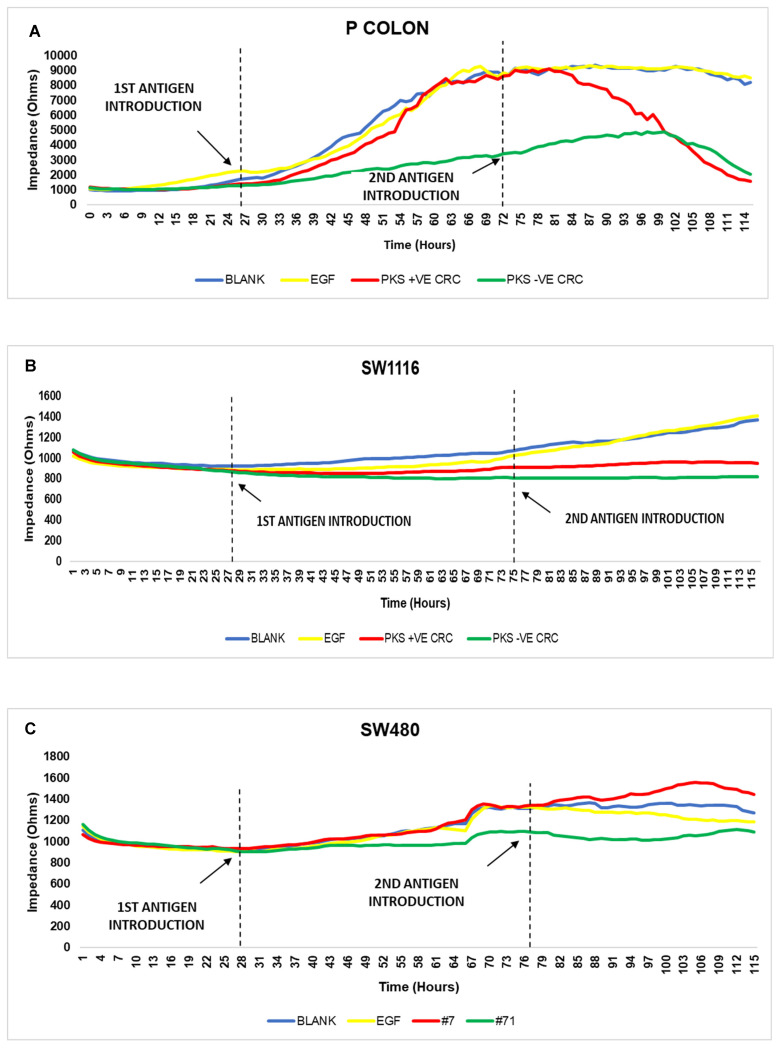

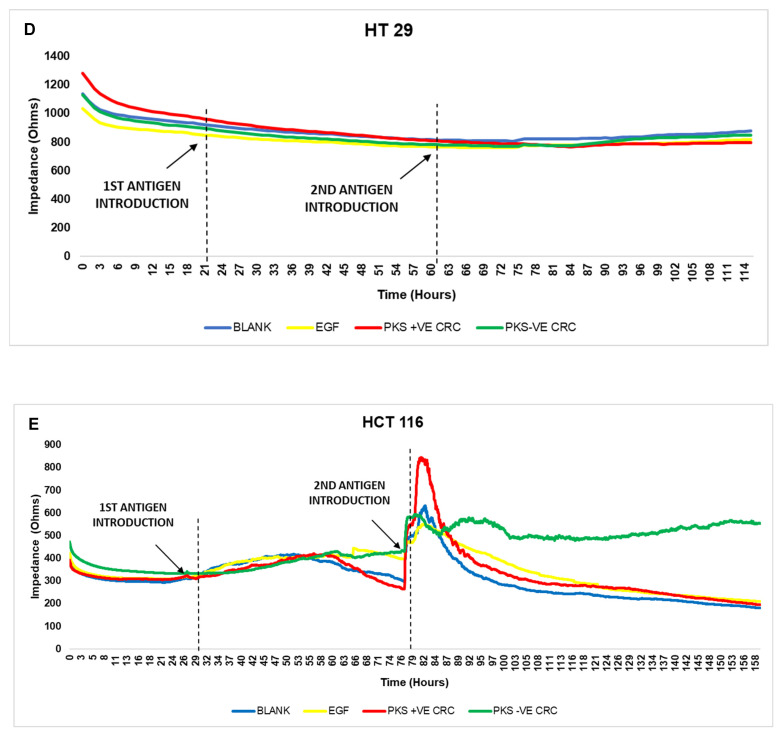
Impedance of CRC cell lines upon recurrent exposure to *K. pneumoniae* antigens. (**A**): healthy, (**B**): stage I, (**C**): stage II, (**D**): stage III, (**E**): stage IV.

The ECIS array aids in detecting an increase in cell proliferation by measuring the impedance of the cell when an alternating current is applied to it. As the cell proliferates and covers the electrodes at the bottom of the well, the impedance increases. Figure 10A–E shows the impedance reading of the CRC cells upon recurrent exposure to *K. pneumoniae* antigens. In this section only, results of the cell lines exposed to PKS +ve CRC and PKS -ve CRC antigens were shown as it had the best enhancement of its group. The enhancement of cell proliferation was seen in all stages of CRC cell lines. Interestingly, the enhancement was more pronounced upon the second exposure of *K. pneumoniae* antigens. Recurrent exposure to *K. pneumoniae* antigens simulates the persistent exposure of the colonic mucosa to bacterial toxins within the colonic microenvironment. The enhancement of cell proliferation due to recurrent infection by *K. pneumoniae* antigens demonstrates the effect that chronic *K. pneumoniae* infection has on cell proliferation and possibly initiation of tumorigenesis. PKS +ve CRC had a higher cell proliferation rate at the early stages of CRC (primary colon, SW1116 and SW480) whereas PKS-ve CRC antigens enhanced cell proliferation at advanced CRC stages (HT29 and HCT116). These findings could potentially illustrate the role of PKS +ve *K. pneumoniae* as a driver bacterium involved in the initiation of tumorigenesis and PKS-ve *K. pneumoniae* as the passenger bacterium that progresses the tumorigenesis into more advanced stages of CRC.

## 4. Discussion

*K. pneumoniae* infections are a huge threat and burden to the healthcare systems as they are the leading cause of nosocomial infections. The emergence of hvKp strains further complicates the treatment and prognosis of a patient. In this study, there was an equal distribution of hvKp strains from PKS-positive and PKS-negative *K. pneumoniae* isolates as well as from CRC and healthy individual origin. Although hvKp strains are generally sensitive to most antibiotics, it is still more difficult to treat compared to cKp infections due to the arsenal of virulence factors that hvKp isolates possess [22]. We observed that all seven MDR strains from this study were isolated from PKS-negative isolates with three isolates being hvKp strains. Additionally, the hvKp-PKS positive isolates screened in this study lacked the genes for resistance towards carbapenems compared to PKS-negative isolates that harboured the bla_OXA-48_ gene. Globally, the K1 and K2 serotype are the most prevalent serotypes of the hvKp strain and PKS-positive *K. pneumoniae* isolates. This could possibly explain the higher rate of antibiotic susceptibility and lack of antibiotic resistance genes observed among PKS-positive strains in this study. The K1 and K2 serotype are known to have increased capsule production that confers the hypermucoviscous variant. This characteristic presents a physical barrier that prevents the horizontal gene transfer of antibiotic resistance genes to occur [23]. However, the concern and focus lies in the ability of cKp strains to inherit and acquire virulence genes from hvKp strains, resulting in a shift from cKp to hvKp [24,25]. There are already increasing reports of MDR-hvKp strains emerging in China and the dissemination and emergence of hvKp strains worldwide should be taken seriously to prevent the further spread of hvKp strains and their related genes [26]. The increase in MDR-hvKp incidence should be further investigated to ascertain if these strains are hvKp strains developing antibiotic resistance or cKp strains acquiring hypervirulent genes from hvKp strains. In addition to this, the increasing rate of PKS-positive *K. pneumoniae* isolated from PLA patients is rampant and should be addressed.

There has been plenty of controversy surrounding the definition of hvKp strains through string test [27]. Hence, other virulence determinants such as the presence of the aerobactin and rmpA gene were used alongside as a more conclusive hvKp identification test. In this study, five out of ten (50%) of the hvKp strains were PKS-positive *K. pneumoniae*, three of which were isolated from CRC patients. Moreover, the characterization of isolates based on virulence genes also showed that these three isolates possessed 22/28 of the virulence genes screened in addition to being a strong biofilm producer and belonging to the K1 serotype. These features aid in increasing their virulence, thus making them strong pathogenic bacterium with the ability to cause life threatening and debilitating diseases within the host.

The key observable difference between the PKS-positive and PKS-negative *K. pneumoniae* isolates is the abundance of virulence genes present within the PKS-positive *K. pneumoniae* genome. PKS-positive isolates had a greater abundance of genes responsible for siderophore production, namely aerobactin, enterobactin, and yersiniabactin, compared to PKS-negative isolates that were more associated with only enterobactin siderophore. The ability to produce more than one group of siderophore increases the organism’s ability to sequester iron more efficiently from the host environment, thus enhancing its survival within the host. A similar observation was made by Khaertynov et al. [28], where aerobactin siderophore was more associated with the presence of the colibactin gene in *rmpA*-positive isolates.

A study by Chen et al. [29] demonstrated that *K. pneumoniae* isolates that harbored a single type of siderophore, particularly enterobactin, had an increased virulence compared to isolates that harbored all four types of siderophore production genes. Similarly, PKS-negative isolates in this study primarily had genes for enterobactin siderophore production only, which translated to having a higher antibiotic resistance thus producing MDR strains. However, this does not diminish the pathogenicity of PKS-positive strains as they are primarily implicated in invasive and persistent infections as well as their increased ability to cause severe infections such as PLA, brain abscess, ophthalmitis, and bacteraemia that results in sepsis [30].

Additionally, the genes coding for allantoin metabolism (*allS, allS2*) was more expressed in PKS-positive strains. Allantoin metabolism is a vital process that allows an organism to utilize allantoin as a source of carbon and nitrogen. Chou et al. [31] showed that *K. pneumoniae* was able to utilize not only carbon but nitrogen as a source of energy under both aerobic and anaerobic conditions. This shows the ability of PKS-positive isolates to sustain and persist under stressful conditions and environments such as the tumour microenvironment within a CRC patient. The results of the virulence gene detection are listed in Supplementary S2.

To further understand if other virulence factors and the origin of isolate may play a synergistic role in PKS-positive isolates, we co-cultured human primary and CRC cell lines derived from stages I-IV with *K. pneumoniae* solubilised antigens obtained from four main isolate groups for 48 h. It is distinctly noticeable that the PKS-positive isolates from CRC origin which are also hvKp strains had the best enhancement of cell proliferation upon exposure to *K. pneumoniae* antigens. PKS-positive isolates, irrespective of healthy or CRC origin, had better enhancement compared to *K. pneumoniae* PKS-negative isolates. The PKS-positive *K. pneumoniae* solubilised antigens contain the genotoxin colibactin which induces DNA double-strand breaks. Furthermore, the solubilised antigens contain certain components such as lipopolysaccharide (LPS) that may induce inflammation resulting in increased cell proliferation. This indicates that the colibactin toxin production along with its counterpart virulence genes seen in the PKS-positive isolates has a better ability to enhance CRC progression compared to PKS-negative isolates. The systematic review conducted by Strakova et al. [32] discussed the possible role of PKS-positive *K. pneumoniae* as a driver bacterium inducing mutations in CRC driver genes. As observed in Figure 9B, PKS-positive isolates of CRC origin showed greater enhancement of cell proliferation upon exposure to *K. pneumoniae* antigens, with the highest proliferation occurring in SW 480 (stage II) CRC-derived cell lines. Contrarily, CRC cell lines exposed to PKS-negative *K. pneumoniae* antigens had a greater enhancement of cell proliferation at advanced CRC stages (stage III-IV). The findings from this study could possibly indicate the driver–passenger relationship between PKS-positive and PKS-negative *K pneumoniae*, respectively. To further explore this notion, an ECIS assay was carried out to substantiate these findings.

The ECIS array was used to confirm the enhancement of CRC cell growth upon exposure to *K. pneumoniae* antigens. Repeated exposure of bacterial antigens increased the enhancement of cell proliferation significantly after the second antigen introduction. This could be due to the medium renewal as well. However, as seen in Figure 10A–E, the medium renewal may have contributed to the enhancement of cell proliferation during the initial stages (primary colon, SW1116 and SW480), however at more advanced stages (stage III and IV) the renewal of medium had little to no effect on cell growth. As observed in Figure 10E, the cells without treatment (blank) had a higher impedance reading compared to the cells exposed to PKS +ve CRC antigens prior to the second antigen introduction. However, after the second antigen introduction, the impedance reading of cells exposed to PKS +ve CRC antigens had a significant spike surpassing the impedance of all others. This demonstrates that the enhancement of cell proliferation was mainly due to the PKS +ve CRC antigens rather than an effect of replenished nutrients. The ECIS study also showed that PKS +ve CRC had a better effect on enhancing cell growth derived from early CRC stages. The impedance reading of these cells showed spikes of cell proliferation enhancement followed by a decrease in impedance, which could be due to the cells achieving 100% confluency within that brief period and thus beginning to detach from the surface of the electrodes. The drastic increase in cell proliferation which is reflected by the spikes of impedance could potentially be caused by an overstimulation of the cells’ signaling pathways. This is necessary as it will trigger the initiation of tumorigenesis thus proving the role of PKS+ve *K. pneumoniae* as a driver bacterium in the initiation and progression of CRC. Another interesting pattern observed here was the ability of PKS-ve CRC-exposed cells to sustain proliferation over longer periods of time compared to PKS+ve CRC-exposed cells. Although we may not see spikes in impedance of cells exposed to PKS-ve CRC cells, they are able to demonstrate a gradual increase in proliferation which will be useful in sustaining the progression of CRC cell proliferation. This is supportive of the notion that PKS-ve *K. pneumoniae* is a passenger bacterium.

**Figure 11:**
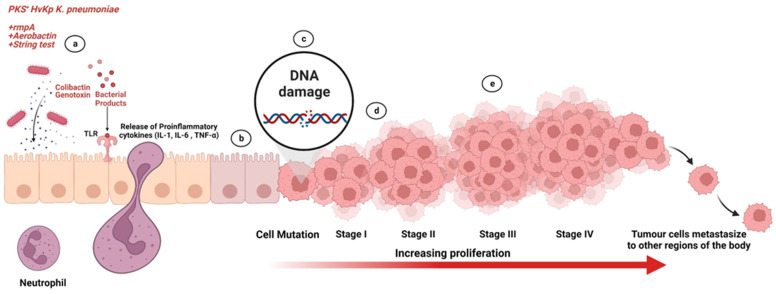
Summary of study depicting action of colibactin genotoxin from HvKp strains on human colonic mucosa. (a) PKS-positive HvKp strain colonizes the host colonic mucosa and exerts damage through the release of colibactin genotoxin. (b) Chronic exposure of colonic mucosa to colibactin genotoxin leads to inflammation of colonic mucosa tissue. (c) Colibactin genotoxin causes DNA double strand breaks in host cells. (d) Mutation occurs in host cells leading in disruption of the genes regulating colonic epithelial cell renewal. (e) Disruption in genes regulating colonic epithelial cell renewal initiates activation and enhancement of tumorigenesis. Illustration created with Biorender.com.

To our knowledge, this is the first known study to highlight the effect of PKS-positive *K. pneumoniae* isolates in possibly enhancing CRC progression in vitro. However, a more in-depth molecular study should be performed to understand the various aspects affected by virulence gene expression during tumorigenesis. Our observation suggests that the presence of the PKS gene and its counterpart virulence genes do play a role in enhancing CRC progression as summarized in Figure 11. Therefore, the emphasis of bacteria screening should include not only the bacterial species but its associated virulence factors. In other words, precautions to prevent further dissemination of MDR, XDR, and hypervirulent (hvKp) *K. pneumoniae* strains is vital to dampen the progression of the tumor while improving the management of CRC patients.

## Figures and Tables

**Table 1:** Antimicrobial categories and agents used.

Antimicrobial Category	Antimicrobial Agent
Aminoglycosides	Amikacin Gentamicin Kanamycin
Antipseudomonal penicillins + β-lactamase inhibitors	Piperacillin-tazobactam Ticarcilin-clavulanic acid
Carbapenems	Imipenem Meropenem
Non-extended spectrum cephalosporins (1st and 2nd generation cephalosporins)	Cefuroxime
Extended spectrum cephalosporins (3rd and 4th generation cephalosporins)	Cefotaxime Ceftazidime Cefepime
Cephamycins	Cefoxitin
Fluroquinolones	Ciprofloxacin
Folate Pathway Inhibitors	Trimethoprim-sulfamethoxazole
Glycylcyclines	Tigecycline
Monobactams	Aztreonam
Penicillins	Ampicillin Amoxycillin Piperacillin
Penicillins + β-lactamase inhibitors	Amoxicillin-clavulanic acid Ampicillin-sulbactam
Phenicols	Chloramphenicol
Polymyxins	Colistin Polymyxin B
Tetracyclines	Doxycycline Tetracycline
Quinolones	Levofloxacin Ofloxacin Norfloxacin

**Table 2:** *K. pneumoniae* presence of PKS colibactin gene and capsular serotype.

Virulence Gene	Colorectal Cancer (n = 15)		Healthy (n = 25)		Total
	PKS +ve	PKS –ve	PKS +ve	PKS -ve	
Colibactin (PKS)	3 (20%)	12 (80%)	7 (28%)	18 (72%)	40
K1 serotype	3	0	0	0	3
K2 serotype	0	1	5	0	6
K5 serotype	0	0	1	2	3
K20 serotype	0	0	0	0	0
K54 serotype	0	0	0	0	0
K57 serotype	0	0	0	2	2
Unknown *	0	11	1	14	26

* The unknown serotypes are the isolates that did not belong to any of the 6 serotypes screened.

**Table 3:** Correlation between Pks gene and serotype against screened virulence genes.

	PKS STATUS	SEROTYPE	wabG	fimH	ycfM	entB	hly	wcaG	ybtS	iutA1	entB1	Aerobactin	IucB	ureA	cnf1	uge	iroN	kfuBC	iroNB	mrkD2	rmpA2	allS	kpn	allS2	iutA	ybtA	rmpA1	kfu	k2A
PKS STATUS	1																												
SEROTYPE	0.335 *	1																											
wabG	0.294	−0.123	1																										
fimH	0.095	0.265	0.163	1																									
ycfM	0.320 *	−0.087	0.946 **	0.138	1																								
entB	0.269	−0.160	0.829 **	0.037	0.780 **	1																							
hly	0.118	−0.145	−0.089	−0.547 **	−0.076	−0.352 *	1																						
wcaG	0.028	−0.250	−0.173	−0.284	−0.241	0.011	0.076	1																					
ybtS	0.620 **	0.336 *	0.141	0.128	0.085	0.092	0.006	0.120	1																				
iutA1	0.569 **	0.386 *	0.191	0.171	0.120	0.155	0.063	0.201	0.373 *	1																			
entB1	0.014	0.210	0.306	0.794 **	0.283	0.150	−0.689 **	−0.283	0.156	0.079	1																		
Aerobactin	0.346 *	0.486 **	0.244	0.402 **	0.201	0.068	−0.063	0.013	0.437 **	0.472 **	0.262	1																	
IucB	0.454 **	0.469 **	0.191	0.314 *	0.120	0.155	−0.172	0.201	0.373 *	0.895 **	0.250	0.577 **	1																
ureA	−0.261	0.101	0.232	0.382 *	0.220	0.246	−0.698 **	−0.220	−0.162	−0.208	0.481 **	0.208	0.120	1															
cnf1	0.257	−0.164	0.203	0.030	0.225	0.046	0.174	0.035	0.012	0.009	−0.046	0.246	0.009	0.078	1														
uge	0.128	−0.135	0.248	−0.004	0.220	0.278	−0.198	0.053	0.313 *	−0.059	0.069	0.059	0.075	0.348 *	0.223	1													
iroN	−0.170	−0.180	0.191	−0.149	0.202	0.181	−0.064	−0.005	−0.274	−0.213	0.092	0.019	−0.213	0.044	0.334 *	0.127	1												
kfuBC	−0.088	−0.208	0.049	0.007	−0.024	0.011	−0.163	0.458 **	0.223	0.201	0.237	0.120	0.201	0.114	0.035	0.190	−0.005	1											
iroNB	−0.057	0.311 *	−0.227	0.154	−0.203	−0.252	−0.084	0.046	0.382 *	−0.283	0.123	0.283	−0.283	0.059	−0.183	0.169	−0.105	0.203	1										
mrkD2	−0.041	0.180	0.010	0.149	−0.005	0.025	−0.371 *	0.005	0.087	0.019	0.223	0.175	0.213	0.563 **	0.138	0.370 *	0.079	0.202	0.105	1									
rmpA2	0.336 *	0.335 *	0.093	0.266	0.124	0.060	−0.146	0.215	0.230	0.624 **	0.212	0.489 **	0.736 **	0.102	0.224	0.007	−0.181	0.215	−0.240	0.181	1								
allS	0.297	−0.224	0.021	−0.408 **	−0.070	0.116	0.399 **	0.549 **	0.127	0.276	−0.579 **	0.078	0.158	−0.278	0.437 **	0.107	−0.160	0.190	−0.212	−0.059	0.259	1							
kpn	0.146	0.266	0.100	−0.048	0.162	0.255	−0.051	−0.162	−0.220	−0.015	−0.095	0.015	−0.015	−0.126	−0.237	−0.363 *	0.225	−0.584 **	−0.160	−0.225	−0.035	−0.244	1						
allS2	0.217	−0.095	0.282	−0.024	0.298	0.115	0.227	0.429 **	0.010	0.402 **	−0.096	0.314 *	0.402 **	0.065	0.493 **	0.188	−0.116	0.429 **	−0.154	0.116	0.492 **	0.568 **	−0.376 *	1					
iutA	0.454 **	0.469 **	0.191	0.314 *	0.120	0.155	−0.172	0.201	0.373 *	0.895 **	0.250	0.577 **	1.000 **	0.120	0.009	0.075	−0.213	0.201	−0.283	0.213	0.736 **	0.158	−0.015	0.402 **	1				
ybtA	0.493 **	−0.081	0.159	−0.138	0.193	0.350 *	0.076	0.132	0.120	0.201	−0.283	0.227	0.094	−0.220	0.424 **	−0.083	0.193	−0.193	−0.268	−0.193	−0.011	0.429 **	0.260	0.138	0.094	1			
rmpA1	0.310 *	−0.131	0.170	−0.179	0.053	0.007	0.499 **	0.493 **	0.206	0.463 **	−0.288	0.210	0.328 *	−0.348*	0.431 **	0.034	−0.127	0.357 *	−0.169	−0.121	0.278	0.799 **	−0.434 **	0.729 **	0.328 *	0.220	1		
kfu	−0.203	−0.324 *	0.180	−0.051	0.119	0.029	0.028	0.296	0.120	0.042	0.125	0.162	0.042	0.140	0.185	0.140	0.129	0.814 **	0.121	0.249	0.079	0.184	−0.602 **	0.468 **	0.042	−0.119	0.382 *	1	
k2A	0.464 **	0.281	0.191	0.116	0.202	0.181	−0.064	−0.202	0.288	0.370 *	0.092	0.213	0.370 *	0.044	−0.138	0.127	−0.079	−0.202	−0.105	0.079	−0.181	−0.160	0.225	−0.116	0.370 *	0.390 *	−0.127	−0.249	1

*. Correlation is significant at the 0.05 level (2-tailed). **. Correlation is significant at the 0.01 level (2-tailed).

**Table 4:** Antibiotic Susceptibility Testing (AST) of *K. pneumoniae* Clinical Isolates.

	Antimicrobial Agent	AK	SXT	TGC	ATM	AMP	AML	AMC	C	DO	TE	LEV	NOR
Isolates													
Colorectal Cancer	PKS +ve (n = 3)	-	-	-	-	2	3	-	-	-	-	-	-
	PKS-ve (n = 12)	1	3	2	1	10	12	1	5	2	3	2	-
Healthy	PKS +ve (n = 7)	1	-	-	-	4	7	-	-	-	-	-	-
	PKS-ve (n = 18)	-	1	-	1	17	18	-	1	1	1	1	1
Total		2	4	2	2	33	40	1	6	3	4	3	1

AK: amikacin, AMC: amoxycillin-clavulanic acid, AML: amoxycillin, AMP: ampicillin, ATM: aztreonam, C: chloramphenicol, DO: doxycycline, LEV: levofloxacin, NOR: norfloxacin, TE: tetracycline, TGC: tigecycline, SXT: trimethoprim-sulfamethoxazole. All *K. pneumoniae* isolates were susceptible to SAM: ampicillin-sulbactam, FEP: cefepime, CTX: cefotaxime, FOX: cefoxitin, CAZ: ceftazidime, CXM: cefuroxime, CIP: ciprofloxacin, CST: colistin, CN: gentamicin, IPM: imipenem, MEM: meropenem, TZP: piperacillin-tazobactam, PB: polymyxin B, OFX: ofloxacin, PIP: piperacillin, KAN: kanamycin, TIM: ticarcilin-clavulanic acid. Hence, AST results were not tabulated.

**Table 5:** Presence of carbapenem-resistance genes among *K. pneumoniae* isolates.

*Resistance Gene*	*Colorectal Cancer*		*Healthy*	
	PKS +ve (n = 3)	PKS –ve (n = 12)	PKS +ve (n = 7)	PKS -ve (n = 18)
*blaKPC*	0	0	0	0
*blaNDM-1*	0	0	0	0
*blaOXA-48*	0	1	0	2
*blaIMP*	0	0	0	0
*blaVIM*	0	0	0	0

**Table 6:** Biofilm forming capabilities among *K. pneumoniae* isolates.

*Biofilm Forming* *Capability*	*Colorectal Cancer*		*Healthy*	
	PKS +ve (n = 3)	PKS –ve (n = 12)	PKS +ve (n = 7)	PKS -ve (n = 18)
*Non-Biofilm*	0	4 (33%)	2 (29%)	6 (33%)
*Weak*	0	1 (8%)	1 (14%)	2 (11%)
*Moderate*	1 (33%)	3 (25%)	1 (14%)	1 (6%)
*Strong*	2 (67%)	4 (33%)	3 (43%)	9 (50%)

## Data Availability

The authors confirm that the data supporting the findings in this article are available upon request.

## Supplementary Materials

The following supporting information can be downloaded at: https://www.mdpi.com/article/10.3390/microorganisms11020443/s1.
