# Supplementary material for: Presence of Polyketide Synthase (PKS) Gene and Counterpart Virulence Determinants in Klebsiella pneumoniae Strains Enhances Colorectal Cancer Progression In-Vitro
Source: Microorganisms. 2023 Feb 9;11(2):443. doi: 10.3390/microorganisms11020443 (PMC9965769; doi:10.3390/microorganisms11020443)
Supplement: Supplementary file 1 [file microorganisms-11-00443-s001.zip › microorganisms-2034771-supplementary.pdf]

Supplementary Files

Virulence genes		Primer Sequence	Amplicon size (bp)
**Adhesion**			
Type 3 Adhesin	*mrkD1*	F: TTCTGCACAGCGGTCCC<br>R: GATACCCGGCGTTTTCGTTAC	240
Type 3 Adhesin	*mrkD2*	F: AAGCTATCGCTGTACTTCCGGCA<br>R: GGCGTTGGCGCTCAGATAGG	340
Type 1 Adhesin	*fimH*	F: TGCTGCTGGGCTGGTCGATG<br>R: GGGAGGGTGACGGTGACATC	550
FimH like Adhesin	*kpn*	F: GTATGACTCGGGGAAGATTA<br>R: CAGAAGCAGCCACCACACG	626
**Iron Acquisition (Siderophores)**			
Enterobactin	*entB*	F: ATTTCCTCAACTTCTGGGGC<br>R: AGCATCGGTGGCGGTGGTCA	371
Enterobactin	*entB1*	F: GTCAACTGGGCCTTTGAGCCGTC<br>R: TATGGGCGTAAACGCCGGTGAT	400
Ferric Aerobactin Receptor	*iutA*	F: GGGAAAGGCTTCTCTGCCAT<br>R: TTATTCGCCACCACGCTCTT	920
Ferric Aerobactin Receptor	iutA1	F: GGCTGGACATCATGGGAACTGG<br>R: CGTCGGGAACGGGTAGAATCG	300
Aerobactin	*aerobactin*	F: GCATAGGCGGATACGAACAT<br>R: CACAGGGCAATTGCTTACCT	556
Aerobactin	*iucB*	F: ATGTCTAAGGCAAACATCGT<br>R: TTACAGACCGACCTCCGTGA	948
Yersiniabactin	*ybtA*	F: ATGACGGAGTCACCGCAAAC<br>R: TTACATCACGCGTTTAAAGG	960
Yersiniabactin	ybtS	F: GACGGAAACAGCACGGTAAA<br>R: GAGCATAATAAGGCGAAAGA	242
Salmochelin Catecholate Receptor	*iroN*	F: AAGTCAAAGCAGGGGTTGCCCG<br>R: GACGCCGACATTAAGACGCAG	665
Salmochelin Catecholate Receptor	*iroNB*	F: GGCTACTGATACTTGACTATTC<br>R: CAGGATACAATAGCCCATAG	992
**Iron Uptake Systems**			
Klebsiella Ferric Iron Uptake	*kfu*	F: GGCCTTTGTCCAGAGCTACG<br>R: GGGTCTGGCGCAGAGTATGC	638
Klebsiella Ferric Iron Uptake	*kfuBC*	F: GAAGTGACGCTGTTTCTGGC<br>R: TTTCGTGTGGCCAGTGACTC	797
**Genotoxins**			
Colibactin	*ClbB*	F: GATTTGGATACTGGCGATAACCG<br>R: CCATTTCCCGTTTGAGCACAC	579
Haemolysin	*hlyA*	F: AACAAGGATAAGCACTGTTCTGGCT<br>R: ACCATATAAGCGGTCATTCCCGTCA	1177
Cytotoxic Necrotising Factor	*cnf*	F: AAGATGGAGTTTCCTATGCAGGAG<br>R: CATTCAGAGTCCTGCCCTCATTATT	498

Capsule synthesis

K2 serotype capsule synthesis	*k2A*	F: CAACCATGGTGGTCGATTAG	531
		R: TGGTAGCCATATCCCTTTGG	
Fucose synthesis for capsule	*wcaG*	F: GGTTGGKTCAGCAATCGTA	169
		R: ACTATTCCGCCAACTTTTGC	
Lipid Polysaccharide (LPS) Biosynthesis	*wabG*	F: ACCATCGGCCATTTGATAGA	683
		R: CGGACTGGCAGATCCATATC	
Outer Membrane Lipoprotein	*ycfM*	F: ATCAGCAGTCGGGTCAGC	160
		R: CTTCTCCAGCATTCAGCG	
Uridine diphosphategalacturonate4-epimerase	*uge*	F: TCTTCACGCCTTCCTTCACT	535
		R: GATCATCCGGTCTCCCTGTA	
Regulator Mucoid Phenotype A	*rmpA1*	F: ACTGGGCTACCTCTGCTTCA	536
		R: CTTGCATGAGCCATCTTTCA	
Regulator Mucoid Phenotype A	*rmpA2*	F: CATAAGAGTATTGGTTGACAG	461
		R: CTTGCATGAGCCATCTTTCA	
Urease synthesis	*ureA*	F: GCTGACTTAAGAGAACGTTATG	337
		R: GATCATGGCGCTACCT(C/T)A	
Allantoin Metabolism	*allS*	CCGAAACATTACGCACCTTT	508
		ATCACGAAGAGCCAGGTCAC	
Allantoin Metabolism	*allS2*	CATTACGCACCTTTGTCAGC	764
		GAATGTGTCGGCGATCAGCTT	

Capsular Polysaccharide

Universal *K. pneumoniae*	*K. pneumoniae Pr1*	F: TTCACTCTGAAGTTTTCTTGTGTTC	130
	K. pneumoniae Pf	R: ATTTGAAGAGGTTGCAAACGAT	
K1 Serotype	*K1-magA*	F: GGTGCTCTTTACATCATTGC	1283
		R: GCAATGGCCATTTGCGTTAG	
K2 Serotype	*K2wzy*	F: GACCCGATATTCATACTTGACAGAG	641
		R: CCTGAAGTAAAATCGTAAATAGATGGC	
K5 Serotype	*K5wzx*	F: TGGTAGTGATGCTCGCGA	280
		R: CCTGAACCCACCCCAATC	
K20 Serotype	*wzyK20*	F: CGGTGCTACAGTGCATCATT	741
		R: GTTATACGATGCTCAGTCGC	
K54 Serotype	*wzxK54*	F: CATTAGCTCAGTGGTTGGCT	881
		R: GCTTGACAAACACCATAGCAG	
K57 Serotype	*wzyK57*	F: CTCAGGGCTAGAAGTGTCAT	1037
		R: CACTAACCCAGAAAGTCGAG	

Carbapenem Resistance

KPC	*blaKPC*	F: CGTCTAGTTCTGCTGTCTTG	798
		R: CTTGTCATCCTTGTTAGGCG	
NDM-1	*blaNDM-1*	F: GGTTTGGCGATCTGGTTTTC	621
		R: CGGAATGGCTCATCACGATC	
OXA-48	*blaOXA-48*	F: GCGTGGTTAAGGATGAACAC	438
		R: CATCAAGTTCAACCCAACC	
IMP	*blaIMP*	F: GGAATAGAGTGGCTTAAYTCTC	232
		R: GGTTTAAYAAAACAACCACC	
VIM	*blaVIM*	F: GATGGTGTTTGGTCGCATA	390
		R: CGAATGCGCAGCACCAG	

Supplementary S1: List of Primers used in this study

Virulence genes	PKS-positive (n=10)		PKS-negative (n=30)		Total
	CRC	Non-CRC	CRC	Non-CRC	
Colibactin	3	7	12	18	40
Hly	0	1	1	0	2
cnf-1	3	0	2	2	7
K1 serotype	3	0	0	0	3
K2 serotype	0	5	1	0	6
K5 serotype	0	1	0	2	3
K20 serotype	0	0	0	0	0
K54 serotype	0	0	0	0	0
K57 serotype	0	0	0	2	2
Unknown	0	1	11	14	26
blaKPC	0	0	0	0	0
blaNDM-1	0	0	0	0	0
blaOXA-48	0	0	1	2	3
blaIMP	0	0	0	0	0
blaVIM	0	0	0	0	0
k_2A	0	3	0	0	3
wcaG	3	1	8	2	14
wabG	3	6	6	13	28
ycfM	3	6	5	12	26
Uge	3	6	11	13	33
rmpA-1	3	1	2	1	7
rmpA-2	3	2	3	3	11
urea	3	6	12	18	39
allS	3	1	4	1	9
allS-2	3	0	3	0	6

Kfu	3	0	8	7	18
kfuBC	3	0	7	4	14
entB	3	6	6	13	28
entB1	3	6	9	18	36
iutA	3	5	3	4	15
iutA1	3	6	2	4	15
Aerobactin	3	6	6	10	25
IucB	3	6	3	4	16
ybtA	3	4	2	4	13
ybtS	3	7	3	6	19
iroN	0	0	1	2	3
iroNB	0	1	2	2	5
mrkD-1	0	0	0	0	0
mrkD-2	3	6	12	16	37
fimH	3	6	8	17	34
Kpn	0	7	6	11	24

Supplementary S2: Prevalence of virulence genes among *K. pneumoniae* clinical isolates.
